# The Effects of Genetic Variation in *FTO* rs9939609 on Obesity and Dietary Preferences in Chinese Han Children and Adolescents

**DOI:** 10.1371/journal.pone.0104574

**Published:** 2014-08-11

**Authors:** Min Yang, Yuyang Xu, Li Liang, Junfen Fu, Feng Xiong, Geli Liu, Chunxiu Gong, Feihong Luo, Shaoke Chen, Chunxiao Xu, Dandan Zhang, Zhengli Li, Shuai Zhang, Yan Zhang, Hao Wang, Yimin Zhu

**Affiliations:** 1 Department of Nutrition, Zhejiang University School of Public Health, Hangzhou, China; 2 Department of Epidemiology & Biostatistics, Zhejiang University School of Public Health, Hangzhou, China; 3 Hangzhou Center for Disease Control and Prevention, Hangzhou, China; 4 Department of Pediatrics, the First Affiliated Hospital of College of Medicine, Zhejiang University, Hangzhou, China; 5 Department of Endocrinology, Children's Hospital of College of Medicine, Zhejiang University, Hangzhou, China; 6 Department of Endocrinology, Children's Hospital Affiliated to Chongqing Medical University, Chongqing, China; 7 Department of Pediatrics, General Hospital of Tianjin Medical University, Tianjin, China; 8 Beijing Children's Hospital Affiliated to Capital Medical University, Beijing, China; 9 Department of Pediatric Endocrinology and Inborn Metabolic Diseases, Children's Hospital of Fudan University, Shanghai, China; 10 Department of Pediatrics Endocrinology, Maternal and Child Health Hospital of Guangxi Zhuang Autonomous Region, Nanning, China; 11 Department of Pathology, Zhejiang University School of Medicine, Hangzhou, China; University of Pennsylvania Perelman School of Medicine, United States of America

## Abstract

The association of the rs9939609 single nucleotide polymorphism in *FTO* gene with obesity has been extensively investigated in studies of populations of European, African, and Asian ancestry. However, inconsistent results have been reported in Asian populations, and the relationship of *FTO* variation and dietary behaviors has only rarely been examined in Chinese children and adolescents. The aim of this study was to assess the association of rs9939609 with obesity and dietary preferences in childhood in a Chinese population. Epidemiological data including dietary preferences were collected in interviews using survey questionnaires, and rs9939609 genotype was determined by real-time PCR. The associations of rs9939609 genotypes with obesity and dietary preferences were analyzed by multivariate logistic regression using both additive and dominant models. The results showed that subjects with a TA or AA genotype had an increased risk of obesity compared with the TT participants; the odds ratios (ORs) were 1.47 (95% CI: 1.25–1.71, *P* = 1.73×10^−6^), and 3.32 (95% CI: 2.01–5.47, *P* = 2.68×10^−6^), respectively. After adjusting for age and gender, body mass index, waist circumference, hip circumference, systolic blood pressure, diastolic blood pressure, fasting blood glucose, triglycerides, and low-density lipoprotein cholesterol were higher, and high-density lipoprotein cholesterol was lower in TA and AA participants than in those with the TT genotype. After additionally controlling for body mass index, the association remained significant only for systolic blood pressure (*P* = 0.005). Compared with TT participants, those with the AA genotype were more likely to prefer a meat-based diet (OR = 2.81, 95% CI: 1.52–5.21). The combined OR for obesity in participants with TA/AA genotypes and preference for a meat-based diet was 4.04 (95% CI: 2.8–5.81) compared with the TT participants who preferred a plant-based diet. These findings indicate the genetic variation of rs9939609 is associated with obesity and dietary preferences in Chinese children and adolescents.

## Introduction

Childhood and adolescent obesity is increasing rapidly in both developed and developing countries [1]–[3]. Over the past 25 years, obesity rates have increased approximately twofold in the US, threefold over 10 years in England, and 3.9-fold over 18 years in Egypt [4]. In China, the prevalence of overweight and obese children and adolescents increased from 5.2% in 1991 to 13.2% in 2006 [5]. Current estimates of obesity rates are higher in developed countries than in developing countries. However, the numbers of obese individuals are much larger in the developing countries [6], [7]. Childhood obesity poses a serious challenge to society because it is associated with the risk of adult obesity and developing obesity-related chronic diseases such as type 2 diabetes mellitus and cardiovascular disease [2], [7], [8]. Thus, more attention should be paid to preventing childhood obesity, especially in developing countries such as China.

Individual susceptibility to obesity is determined by interactions between genetic and environmental factors [9], [10]. The fat mass and obesity associated (*FTO*) gene was the first obesity-related gene discovered by large-scale genome-wide association studies (GWAS), although its functional implications still need further validation [11]–[15]. Since the *FTO* gene was first identified in 2007, many subsequent studies have also demonstrated an association of *FTO* genetic variants with obesity and body mass index (BMI) in various ethnic populations in both children and adults [16]–[22]. The *FTO* rs9939609 single nucleotide polymorphism (SNP) variant is of particular interest because it has the strongest known effect on increased BMI [12], and its association with BMI and obesity-related phenotypes has been confirmed by independent studies in large Caucasian populations [16], [17].

However, studies of this association in Asian populations or child populations have yielded inconsistent results. Cha et al. reported a significant association of *FTO* genetic variants with BMI in a Korean population in 2008 [23]. Subsequently, a growing number of studies, including a few literature-based meta-analyses, have provided more evidence that the rs9939609 variant may be associated with BMI in east and south Asians, including Chinese, Japanese, Vietnamese, Malaysian, and Indians [9], [22]–[35]. However, the first study performed in Mainland China, in a population of 3,210 Chinese Han adults 50 to 70 years of age, found no association of *FTO* variants (rs9939609, rs8050136, and rs9930506) with BMI, waist circumference (WC), or other obesity-related traits [36]. Similarly, Horikoshi et al. found no association of *FTO* variants with BMI in a Japanese population [37]. Available, but limited evidence suggests that the association between rs9939609 and BMI in children changes with age [38]–[40]. A recent study conducted in a large sample of preschool children in Europe found no relationship between rs9939609 and BMI [41]. In another large meta-analysis, carriers of the *FTO* rs9939609 A allele were found to have a lower body weight before the age of 2.5 than carriers of the common allele [42]. Few studies have been conducted to examine the relationship of *FTO* variants with obesity in Asian children and adolescents, particularly in large childhood populations [18], [43], [44].

Evidence to date suggests that the association between rs9939609 variations and BMI may be predominantly driven by increased energy intake, particularly fat consumption and impaired satiety [45]–[49]. However, the relationships of rs9939609 with dietary intake and obesity found in recent studies have been inconsistent [46]–[53]. A population-based survey carried out in school-age children residing in Beijing showed that protein-rich foods, vegetables, and fruits may modify the effects of *FTO* rs9939609 variant on the risk of childhood obesity [54]. In addition, a recent study conducted in a selected cohort of obese children from a genetic isolate population in Sardinia found that the rs9939609 variant was associated with BMI but did not influence eating behavior, neither in the entire cohort nor when participants were stratified by age [55]. The findings from another study using a multi-ethnic population in Canada indicated that the rs9939609 variant was associated with intake of dietary macronutrients for Canadian aboriginals and Europeans only, but not in ethnic Chinese [56]. Traditional dietary patterns and preferences in China, especially among children and adolescents, are currently undergoing substantial changes. Therefore, further studies are needed to evaluate the interactions between rs9939609 variants and dietary preferences that influence obesity in China, especially in populations of children and adolescents.

The purpose of the present study was to examine the association of the *FTO* rs9939609 polymorphism with the risk of obesity and obesity-related traits in children and adolescents from different areas of China. The study also examined the relationship between rs9939609 and dietary preferences.

## Materials and Methods

### Subjects

A cross-sectional study on metabolic syndrome with cluster sampling was conducted in six cities in China (Beijing, Tianjin, Chongqing, Hangzhou, Shanghai and Nanning) in 2010. All students from 7 to 18 years of age in twelve selected schools were eligible to participate in this survey. Students with chronic heart, lung, liver and renal diseases, cancer, and other serious illnesses were excluded. After excluding survey questionnaires with incomplete or suspected incorrect answers, a final evaluable sample of 16,580 participants (8,477 boys and 8,103 girls) was obtained.

**Figure 1:**
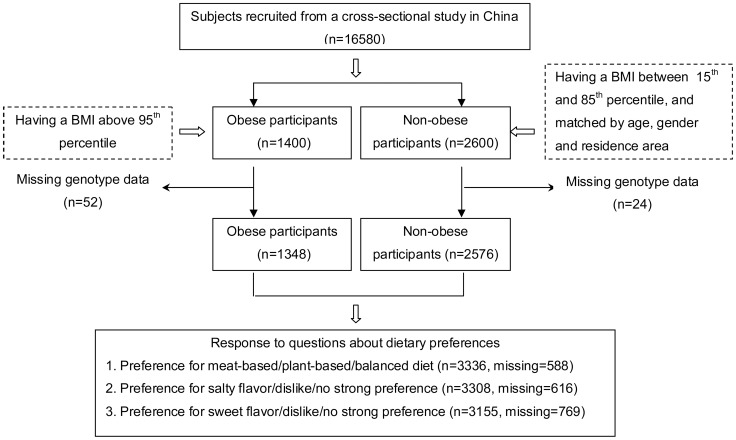
Participant selection and disposition.

A total of 1400 obese students (950 boys and 450 girls) and 2600 non-obese control students (1700 boys and 900 girls) were randomly selected from the survey population. Obesity was defined as a BMI above the 95^th^ percentile of the Chinese BMI reference data for Han children and adolescents in specific age- and gender- groups [57]. The control students had BMIs between the 15^th^ and 85^th^ percentile and were matched by age, gender and residence area to the obese students. Individuals with missing genotype data were excluded, leaving 1348 obese and 2576 control students who were enrolled in this study. The study protocols were approved by the research ethics committees of Zhejiang University and all collaborating hospitals. All the participants and their guardians provided written informed consent. A study flowchart, including information on missing data, is provided in Figure 1. The BMI thresholds of the 15^th^, 85^th^, 95^th^ percentiles for Chinese Han children and adolescents in specific age- and gender- groups are shown in Table S1.

### Anthropometric measurements and epidemiologic investigation

Anthropometric measurements, including height, weight, waist circumference (WC) and hip circumference (HC), systolic blood pressure (SBP) and diastolic blood pressure (DBP), were measured by trained physicians or investigators, following a standard protocol. Height (to the nearest 0.1 cm) and weight (to the nearest 0.1 kg) were measured with the participants wearing light clothing and without shoes. BMI was calculated as body weight in kilograms divided by the square of height in meters (kg/m^2^). WC (to the nearest 0.1 cm) was measured at the midpoint between the iliac crest and the lower costal margin while standing and at the end of an exhalation. HC (to the nearest 0.1 cm) was measured while standing at the maximum circumference around the buttocks. After at least a 5-minute rest period, blood pressure was measured in a sitting position with a mercury sphygmomanometer. The SBP and DBP were reported as the average of three repeat measurements with 30-second rest intervals between measurements.

After a 12-hour overnight fast, blood samples were drawn to determine the serum levels of total cholesterol (TC), total triglyceride (TG), low-density lipoprotein cholesterol (LDLC), high-density lipoprotein cholesterol (HDLC) and fasting blood glucose (FG) with a biochemical autoanalyzer (Hitachi 7060, Tokyo, Japan).

Demographic characteristics, health status, dietary preferences, physical activity, medical history and family history of obesity were collected using a standardized questionnaire during a face-to-face interview by a trained investigator. The dietary data included participants' preference for type of diet (meat-based, plant-based or balanced diet), preference for salty flavor (like, dislike or no strong preference), and preference for sweet flavor (like, dislike or no strong preference). The questionnaire was administered to 58 randomly selected participants from Hangzhou prior to the formal interview. Test-retest reliability was assessed by re-administering the questionnaire 4 weeks later, and validity was assessed by comparing 3-day food records. The reliability (Cronbach's α) and content validity of the questionnaire were 0.86 and 0.88, respectively.

### Genotyping

Genomic DNA was extracted from peripheral blood, using the TOYOBO MagExtractor Genomic DNA Purification Kit (Toyobo, Osaka, Japan) following the protocol recommended by the vendor. The rs9939609 polymorphisms were genotyped using a TaqMan-based SNP allelic genotyping assay (Applied Biosystems, Foster City, USA). One-hundred-and-fifty replicate quality control samples were included in the genotyping plates with more than 99% concordance (149/150).

### Statistical analysis

Summary statistics were performed to describe the characteristics of study participants, which were stratified by case/control status. The quantitative data were expressed as means ± standard deviations (SD) for approximately normal distributions, as medians (inter-quartile range) for non-normally distributed variables, and as frequencies (percentages) for categorical variables. Non-normally distributed variables were square-root transformed before analysis. The information on dietary preferences was analyzed as dichotomies of diet preference (+, meat-based diet; −, plant-based or balanced diet), salty flavor (+, like; −, dislike/no strong preference), and sweet flavor (+, like; −, dislike/no strong preference). Participants with missing genotype data were excluded from the analysis.

The Hardy–Weinberg equilibrium of rs9939609 was examined in control students with Pearson's chi-squared test. The *t*-test was used to compare the mean differences between obese and control students for normally distributed variables, and the Kruskal–Wallis test was used for non-normally distributed variables. The chi-square test was used to compare the frequency of differences in categorical variables. Associations of rs9939609 with obesity and dietary preferences were analyzed with multivariate logistic regression using both additive (TT/TA/AA) and dominant (TT/TA+AA) models. The interaction effects for obesity between rs9939609 and dietary preferences were calculated with a logistic regression model adding an additional interaction term. Comparisons of obesity-associated traits were carried out using multivariate linear regression that included covariates such as age, gender and BMI. All the analyses were performed using SPSS for Windows, version 16.0. A *P*-value <0.05 was considered to be statistically significant.

## Results

### Subject characteristics

The demographic and biochemical data for the total of 3924 evaluated participants (excluding 76 with missing genotype data) are shown in Table 1. The mean age ± SD was 11.0±2.6 years in the obese group and 11.6±2.4 years in the control group (*P*<0.0001). The percentage of males was 68.8% in the case group and 66.0% in the control group (*P* = 0.07). The obese participants had significantly higher levels of BMI, WC, HC, SBP, DBP, FG, TG, TC and LDLC, but a significantly lower level of HDLC than the controls (all *P*<0.0001).

**Table 1:** Characteristics of the obese and control participants.

Variables	Obese group		Control group		Total&		*P-*value*
	n	Mean/median	n	Mean/median	n	Mean/median	
Gender (n, %)							0.07^b^
Male	928 (68.8%)		1699 (66.0%)		2627 (67.0%)		
Female	420 (31.2%)		877 (34.0%)		1297 (33.0%)		
Age (years)	1348	11.0±2.6	2576	11.6±2.4	3924&	11.4±2.5	<0.0001^a^
BMI (kg/m^2^)	1348	27.2±4.0	2576	17.0±2.3	3924&	20.5±5.7	<0.0001^a^
Waist circumference (cm)	1338	85.0±12.4	2565	60.5±8.2	3903	68.9±15.2	<0.0001^a^
Hip circumference (cm)	1243	92.0±12.1	2562	73.1±10.6	3805	79.3±14.2	<0.0001^a^
Systolic blood pressure (mm Hg)	1342	112.8±13.8	2556	104.2±11.5	3898	107.1±13.0	<0.0001^a^
Diastolic blood pressure (mm Hg)	1340	68.4±9.9	2556	63.9±8.0	3896	65.4±8.8	<0.0001^a^
Fasting blood glucose (mmol/L)	1335	5.0 (4.5–5.2)	2573	4.6 (4.2–5.0)	3908	4.7 (4.3–5.1)	<0.0001^c^
Total triglyceride (mmol/L)	1250	1.1 (0.8–1.6)	2574	0.7 (0.6–0.9)	3824	0.8 (0.6–1.1)	<0.0001^c^
Total cholesterol (mmol/L)	1307	4.2 (3.7–4.7)	2574	3.8 (3.4–4.3)	3881	3.9 (3.5–4.4)	<0.0001^c^
LDLC (mmol/L)	1296	2.4 (2.0–2.8)	2574	1.9 (1.6–2.3)	3870	2.0 (1.7–2.5)	<0.0001^c^
HDLC (mmol/L)	1296	1.2 (1.1–1.5)	2574	1.5 (1.3–1.7)	3870	1.4 (1.2–1.6)	<0.0001^c^

& Excluding missing genotype data in 76 participants (52 obese and 24 controls).

*P-values for differences in distribution of characteristics between the obese and control groups.

Fasting blood glucose, total triglyceride, total cholesterol, LDLC, HDLC are expressed as median (lower quartile–upper quartile); other variables are expressed as mean ±SD.

a Independent *t*-test;

b χ^2^ test;

c Kruskal–Wallis test.

Abbreviations: BMI, body mass index; LDLC, low-density lipoprotein cholesterol; HDLC, high-density lipoprotein cholesterol.

### *FTO* rs9939609 and obesity

The frequency of variant allele (A) was 11.1% in the control group and 16.3% in the obese group. This polymorphism was in Hardy–Weinberg equilibrium in the control group (*P*>0.05). Table 2 shows the association between rs9939609 polymorphism and obesity in the study subjects. The A allele of rs9939609 was significantly associated with an increased risk for obesity with an OR of 1.56 (95% CI: 1.36–1.78, *P = *2.24×10^−10^), which means each additional copy of the A allele represented a 1.56-fold increase in the risk of obesity. Compared with the homozygous wild-type (TT) participants, heterozygous (TA) participants had a greater risk of obesity, with an OR of 1.47 (95% CI: 1.25–1.71, *P* = 1.73×10^−6^), and those who were homozygous for the variant allele (AA) had an OR of 3.32 (95% CI: 2.01–5.47, *P* = 2.68×10^−6^). Under the dominant model, the participants with the A allele had increased risk of obesity, with an OR of 1.56 (95% CI: 1.34–1.81, *P* = 1.19×10^−8^).

**Table 2:** Association of the *FTO* rs9939609 genotype and obesity.

rs9939609	Obese group	Control group	Additive model		Dominant model	
	n (%)	n (%)	*P-*value^§^	OR (95% CI)	*P-*value*	OR (95% CI)
**TT**	951 (70.5)	2031 (78.8)	9.62×10^−11^	1.00		1.00
**TA**	356 (26.4)	519 (20.1)		1.47 (1.25–1.71)	1.19×10^−8^	1.56 (1.34–1.81)
**AA**	41 (3.0)	26 (1.0)		3.32 (2.01–5.47)		

Abbreviations: OR, odds ratio; CI, confidence interval.

*P-*value was calculated with Logistic regression using additive model^§^, and dominant model* adjusted for age and gender.

### *FTO* rs9939609 and obesity-related metabolic traits

Linear regression models were used to estimate the associations between *FTO* rs9939609 and obesity-related metabolic traits in the additive model (Table 3). After adjusting for age and gender, BMI (*P* = 9.53×10^−11^), WC (*P* = 4.22×10^−10^), HC (*P* = 5.49×10^−7^), SBP (*P* = 2.12×10^−7^), DBP (*P* = 0.008), TG (*P* = 0.045), LDLC (*P* = 0.027), and FG (*P* = 0.012) were higher, and HDLC (*P* = 0.001) was lower, in the participants with TA or AA than in participants with the TT genotype. After further adjustment for BMI, the only association that remained significant was with SBP (*P* = 0.005).

**Table 3:** Association of the *FTO* rs9939609 genotypes with clinical and metabolic measurements.

Quantitative trait	TT		TA		AA		*P-*value^ a^	*P* value^ b^
	n	Mean/median	n	Mean/median	n	Mean/median		
BMI (kg/m^2^)*	2982	20.2±5.6	875	21.3±5.9	67	23.1±5.4	9.53×10^−11^	–
Waist circumference (cm) *	2966	68.2±15.1	872	70.9±15.7	65	75.7±14.1	4.22×10^−10^	0.449
Hip circumference (cm) *	2900	78.8±14.1	841	80.7±14.5	64	83.2±15.0	5.49×10^−7^	0.549
Systolic blood pressure (mmHg) *	2964	106.7±13.0	869	108.3±13.0	65	113.3±14.9	2.12×10^−7^	0.005
Diastolic blood pressure (mmHg) *	2962	65.3±8.7	869	65.9±9.0	65	67.7±10.0	0.008	0.423
Fasting blood glucose (mmol/L)#	2968	4.7 (4.3–5.1)	873	4.7 (4.3–5.2)	67	4.9 (4.4–5.2)	0.012	0.242
Total triglyceride (mmol/L) #	2913	0.8 (0.6–1.1)	847	0.8 (0.6–1.2)	64	0.8 (0.6–1.3)	0.045	0.929
Total cholesterol (mmol/L) #	2950	3.9 (3.5–4.4)	866	4.0 (3.5–4.4)	65	3.9 (3.5–4.3)	0.330	0.694
LDLC (mmol/L) #	2942	2.0 (1.7–2.4)	862	2.1 (1.7–2.5)	66	2.0 (1.7–2.4)	0.027	0.942
HDLC (mmol/L) #	2943	1.4 (1.2–1.7)	862	1.4 (1.2–1.6)	65	1.4 (1.1–1.5)	0.001	0.106

*Data are expressed as mean±SD;

# Data are expressed as medians (lower quartile–upper quartile) and are square root transformed before regression analysis.

*P-*value was calculated with Linear regression using additive model, ^a^: adjusted for age and gender; ^b^: adjusted for age, gender and BMI.

Abbreviations: BMI, body mass index; LDLC, low-density lipoprotein cholesterol; HDLC, high-density lipoprotein cholesterol.

### *FTO* rs9939609 and dietary preferences

The dietary preferences in different genotypes of rs9939609 are described in Table 4. 36.2% of the participants with the AA genotype preferred a meat-based diet, which was higher than participants with TA (15.4%) and TT (14.5%) genotypes. After adjusting for age, gender, and BMI in the logistic regression model, more AA than TT participants preferred a meat-based diet (OR = 2.81, 95% CI: 1.52–5.21; *P* = 0.001). No significant associations were found for preference of sweet or salty foods (all *P*>0.05).

**Table 4:** Associations of the *FTO* rs9939609 genotypes with dietary preferences.

*FTO* Genotype	Dietary preference&, n (%)		OR (95% CI)	*P* value#
	−	+		
	**Prefer plant-based diet**	**Prefer meat-based diet**		
	**/prefer balanced diet**			
TT	2195 (85.5)	372 (14.5)	1.00	−
TA	611 (84.6)	111 (15.4)	1.01 (0.80–1.28)	0.913
AA	30 (63.8)	17 (36.2)	2.81 (1.52–5.21)	0.001
	**Dislike salty flavor**	**Like salty flavor**		
	**/no strong preference**			
TT	2194 (86.1)	355 (13.9)	1.00	−
TA	603 (84.7)	109 (15.3)	1.07 (0.84–1.36)	0.578
AA	39 (83.0)	8 (17.0)	1.08 (0.50–2.37)	0.840
	**Dislike sweet flavor**	**Like sweet flavor**		
	**/no strong preference**			
TT	851 (35.2)	1568 (64.8)	1.00	−
TA	242 (35.0)	450 (65.0)	1.01 (0.84–1.20)	0.959
AA	10 (22.7)	34 (77.3)	1.84 (0.90–3.76)	0.097

# Logistic regression, adjusted for age, gender and BMI.

& Diet preference: +, meat-based diet; −, plant-based diet or balanced diet. Salty flavor: +, like; −, dislike / no strong preference; Sweet flavor: +, like; −, dislike / no strong preference.

Abbreviations: OR, odds ratio; CI, confidence interval.

### *FTO* rs9939609, dietary preferences and obesity

No multiplicative interactions (all *P*>0.05) between *FTO* rs9939609 and dietary preferences on obesity were found, as described in Table 5. Compared with TT participants who did not prefer a meat-based diet, the OR for obesity in the participants with TA/AA genotypes and preferred a meat-based diet was 4.04 (95% CI: 2.80–5.81, *P* = 4.88×10^−14^). Similar results were observed for the effects of the rs9939609 TA and AA genotypes combined with salty flavor preference (OR = 3.31, 95% CI: 2.26–4.84, *P* = 1.33×10^−10^) and sweet flavor preference (OR = 1.42, 95% CI: 1.10–1.83, *P* = 0.006).

**Table 5:** Joint effects between the *FTO* rs9939609 genotypes, obesity, and dietary preferences.

Genotype	Dietary preference	Obesity (n, %)	Control (n, %)	OR (95% CI)	*P*-value#
	Meat-based diet preference a				
TT	−	454 (54.2)	1741 (69.7)	1	−
TA + AA	−	169 (20.2)	472 (18.9)	1.37 (1.12–1.68)	0.002
TT	+	149 (17.8)	223 (8.9)	2.56 (2.02–3.23)	2.06×10^−15^
TA+AA	+	66 (7.9)	62 (2.5)	4.04 (2.80–5.81)	4.88×10^−14^
***P*** ** for interaction** *					0.534
	Salty flavor preference b				
TT	−	469 (56.5)	1725 (69.6)	1	−
TA+AA	−	176 (21.2)	466 (18.8)	1.38 (1.13–1.69)	0.001
TT	+	131 (15.8)	224 (9.0)	2.25 (1.77–2.86)	2.43×10^−11^
TA+AA	+	54 (6.5)	63 (2.4)	3.31 (2.26–4.84)	1.33×10^−10^
***P*** ** for interaction** *					0.925
	Sweet flavor preference c				
TT	−	195 (24.7)	656 (27.7)	1	−
TA+AA	−	72 (9.1)	180 (7.6)	1.34 (0.98–1.84)	0.087
TT	+	373 (47.3)	1195 (50.5)	1.01 (0.83–1.23)	0.901
TA+AA	+	148 (18.8)	336 (14.2)	1.42 (1.10–1.83)	0.006
***P*** ** for interaction** *					0.772

a : Meat-based diet preference: +, meat-based diet; −, plant-based diet or balanced diet;

b : Salty flavor preference: +, like; −, dislike/no strong preference;

c Sweet flavor preference: +, like; −, dislike / no strong preference.

# Adjusted for age and gender.

*Logistic regression.

## Discussion

In the present study, *FTO* rs9939609 was found to be significantly associated with the risk of obesity and also with dietary preferences independently of BMI. In addition, rs9939609 was associated with multiple cardiovascular and metabolic risk factors, however after additional adjustment for BMI, only SBP still demonstrated a statistically significant association. This study confirms the association of *FTO* rs9939609 with obesity and dietary preferences in Chinese Han children and adolescents.

Childhood and adolescent obesity has become a serious public health problem. It predisposes individuals to a higher incidence of diseases related to cardiovascular, endocrine, respiratory, and immune function, and greatly increases the risk of chronic diseases in adulthood. Although the pathogenesis of obesity and its associated co-morbidities are multifactorial, increasing evidence has implied that the *FTO* gene, and in particular SNP rs9939609, play an important role in the occurrence and development of obesity in different ethnic populations in both children and adults [11], [12]. The association of the *FTO* rs9939609 variant with both increased BMI and obesity-related phenotypes has been examined by independent studies of large Caucasian populations. However, the data are less conclusive in non-Caucasian and childhood-age populations. A study by Li et al. found no significant association between rs9939609 and obesity in a Chinese Han population [36], but those findings were inconsistent with the results of a subsequent study performed in a Taiwanese population [58]. Hallman et al. found that the A/A genotype of rs9939609 was associated with higher BMI in non-Hispanic whites at all ages, but there were no significant associations in African Americans [19]. A study of 670 Chinese children and adolescents indicated that *FTO* rs9939609 was strongly associated with BMI and obesity-related metabolic traits [18]. However, a study of a relatively large sample size (n = 1718) of European preschool children (3–4 years of age) found no significant association [41]. These inconsistent results might be explained by ethnic differences in the allele frequencies of *FTO*, differences in population samples and age, and differences in the underlying distributions of key environmental exposures. Therefore, the current study was designed to examine the associations of *FTO* rs9939609 with the risk of obesity and obesity-related metabolic traits in 3924 Chinese Han children and adolescents (7–18 years of age) living in the northern, central and southern regions of China. In addition, the influence of rs9939609 on dietary preferences (preference for a meat-based diet, salty tastes, and sweet tastes) was evaluated to try to account for any observed physical and metabolic associations.

In the present study, the minor allele frequency (MAF) of rs9939609 in control participants was 11.1%, which is in line with that reported for Chinese populations in previous studies [18], [36], [58]. However, the risk allele A (mutant allele) of rs9939609 is more common in populations of European origin (MAF 0.45–0.48), which means 69.8% of the populations carry at least one risk allele and 20.3% carry two risk alleles. In this study, results showed that rs9939609 was associated with the risk of childhood obesity and were consistent with the results of two previously published independent studies conducted in Beijing, as mentioned above [18], [59]. However, the association was not statistically significant in a study that had been conducted in Chinese adults [36]. This inconsistency may be explained by the difference in age range of participants. A study by Loos et al. in a European population showed that the association between the *FTO* gene, obesity and BMI was more significant in children than in adults [60]. Qi et al. found that the strength of association between rs9939609 and BMI decreased with age in American male doctors [61]. The researchers speculated that environment-related influences accumulated with age and that this phenomenon may partly explain the reduction in association at an older age. Hence, more studies are needed to validate the age-related differences observed in the association between rs9939609 and obesity, and to clarify the underlying mechanism.

The results of the current study also revealed significant association of rs9939609 with several metabolic risk factors, such as WC, HC, SBP, DBP, FG and HDLC, but not with TG, TC or LDLC, when adjusted for age and gender. However, after adding BMI in the model above, only SBP retained its significant association. These findings imply that the associations of the rs9939609 variation with the above obesity-related traits may be mediated through BMI. Similar results have been reported in Chinese and Japanese populations. However, the results were inconsistent in Europeans [18], [43], [58]. Nevertheless, our study found that rs9939609 was independently associated with SBP, and this finding was consistent with some recent studies [62], [63]. The variant allele (A) of rs9939609 might be an independent risk factor for increasing blood pressure/hypertension, the mechanism remains unknown. Further functional studies are required to clarify this association.

Increasing energy intake is a major contributor to the current obesity epidemic [46]. Evidence to date suggests that the association between rs9939609 and BMI may be predominantly driven by increased energy intake [53]. Cecil et al. reported that the A allele at rs9939609 predisposed to obesity but did not appear to be involved in the regulation of energy expenditure; instead, this allele may have a role in the control of food intake and food choice in European children, suggesting a link to a hyperphagic phenotype or a preference for energy-dense foods [46]. A study in Scottish children found that the rs9939609 obesity risk A allele was significantly associated with increased energy intake independent of body weight [50]. Timpson et al. also found that carriers of the minor variants of rs9939609 consumed more fat and total energy than those non-carriers [47]. Recently, further evidence has suggested that *FTO* interacts with energy intake patterns in children, as related to obesity risk [64]–[68]. The findings of this study showed that the genetic variation of rs9939609 was associated with a meat-based dietary preference, and children with the A allele were predisposed to prefer a meat-based diet, which is characterized by higher energy density, higher fat, and lower dietary fiber than either plant-based or balanced diets. Previous studies also found that *FTO* affected fat cell lipolysis [69], [70]. Therefore, *FTO* might be involved in the incidence of obesity by both direct and indirect mechanisms.

Although no significant multiplicative interactions were observed between rs9939609 and dietary preferences, their joint effects on obesity were observed in this study. As discussed earlier, the reason a meat-based diet predisposes to obesity may be its high energy density. As for strong tastes, the effect on obesity was consistent with a previous study [71]. However, preference for sweets was not significantly associated with obesity in the current study. On the contrary, another study showed a positive correlation between hedonic response (i.e., foods rich in sugar and fat) and weight gain in Pima Indians [72]. This inconsistency may be caused by the heterogeneity of cohorts and differences in the amounts of sweet foods consumed. These results indicate that dietary preferences play an important role in the development of obesity.

The current study has several strengths. First, the study population was a well-defined, homogenous group of children and adolescents, and standardized methods were used to measure the clinical parameters, such as height and weight. Second, the influence of rs9939609 on dietary preferences (preference for a meat-based diet, salty taste, or sweet taste) was examined to explain how variation of rs9939609 confers a predisposition to obesity. Third, the interaction of rs9939609 with three dietary factors was examined as well as their joint effect on obesity. Several limitations, however, are apparent. First, our findings may provide significant implications for Chinese and related ethnic groups, but they may not apply to other ethnicities. Therefore, further evaluations in other ethnic groups are needed. Second, information about dietary preferences was self-reported. The data would be more reliable if food intake were more accurately measured with diet diary records. However, recording a diet diary is difficult in epidemiological surveys with relatively large samples, such as in this study. Third, we adjusted for key covariates such as age, gender and BMI, but other related covariates (i.e., socioeconomic status, pubertal status, and physical activities) were not considered because of the high proportion of missing data. Fourth, the number of AA/AT individuals who had health-risk-related dietary preferences (i.e., meat-based diet, salty or sweet flavor) was very small because of the low frequency of the variant allele (A) in the Chinese population. Hence, further studies with larger populations are needed.

In brief, this study found that the *FTO* rs9939609 variation is significantly associated with the risk of obesity and a meat-based dietary preference in Chinese Han children and adolescents. Further studies are needed to understand the functional mechanisms underlying this association.

## Supporting Information

Table S1 **BMI thresholds (Kg/m^2^) of the 15^th^, 85^th^, 95^th^ percentile for the Chinese Han children and adolescents in specific age- and gender- groups.**
(DOC)
