# Supplementary material for: The Effects of Genetic Variation in FTO rs9939609 on Obesity and Dietary Preferences in Chinese Han Children and Adolescents
Source: PLoS One. 2014 Aug 11;9(8):e104574. doi: 10.1371/journal.pone.0104574 (PMC4128666; doi:10.1371/journal.pone.0104574)
Supplement: Table S1 — BMI thresholds (Kg/m2) of the 15th, 85th, 95th percentile for the Chinese Han children and adolescents in specific age- and gender- groups. (DOC) [file pone.0104574.s001.doc]

**Table S1:** BMI thresholds (Kg/m^2^) of the 15^th^, 85^th^, 95^th^ percentile for the Chinese Han children and adolescents in specific age- and gender- groups

| **Age (years)** | **Boys** | | |  | **Girls** | | |
| --- | --- | --- | --- | --- | --- | --- | --- |
|  | **15%ile** | **85%ile** | **95%ile** |  | **15%ile** | **85%ile** | **95%ile** |
| 7 | 13.4 | 17.4 | 19.2 |  | 13.2 | 17.2 | 18.9 |
| 8 | 13.8 | 18.1 | 20.3 |  | 13.4 | 18.1 | 19.9 |
| 9 | 14.0 | 18.9 | 21.4 |  | 13.7 | 19.0 | 21.0 |
| 10 | 14.3 | 19.6 | 22.5 |  | 14.1 | 20.0 | 22.1 |
| 11 | 14.7 | 20.3 | 23.6 |  | 14.6 | 21.1 | 23.3 |
| 12 | 15.1 | 21.0 | 24.7 |  | 15.2 | 21.9 | 24.5 |
| 13 | 15.7 | 21.9 | 25.7 |  | 15.8 | 22.6 | 25.6 |
| 14 | 16.3 | 22.6 | 26.4 |  | 16.3 | 23.0 | 26.3 |
| 15 | 16.8 | 23.1 | 26.9 |  | 16.7 | 23.4 | 26.9 |
| 16 | 17.3 | 23.5 | 27.4 |  | 16.9 | 23.7 | 27.4 |
| 17 | 17.7 | 23.8 | 27.8 |  | 17.1 | 23.8 | 27.7 |
| 18 | 18.1 | 24.0 | 28.0 |  | 17.2 | 24.0 | 28.0 |
